# Distributional patterns and habitat associations of sturgeon chub in western Missouri River tributaries of South Dakota

**DOI:** 10.1111/jfb.70414

**Published:** 2026-03-29

**Authors:** Mitchell R. Magruder, Jenna P. Ruoss, Mark A. Pegg

**Affiliations:** ^1^ San Antonio River Authority San Antonio Texas USA; ^2^ School of Natural Resources University of Nebraska‐Lincoln Lincoln Nebraska USA

**Keywords:** habitat associations, imperilled species, Missouri River tributaries, sturgeon chub

## Abstract

There is a growing body of literature that suggests riverine fish are some of the most threatened taxa on a global scale. Similarly, the literature suggests less‐altered tributaries may offer refugia for large‐river specialists. The greater Mississippi River basin, including the Missouri River system, has been subjected to anthropogenic changes in flow, habitat fragmentation and turbidity, leading to declines in multiple native fish species. Small‐bodied fish species are exceptionally susceptible to changes in habitat, resulting in a heightened risk of extinction. The sturgeon chub *Macrhybopsis gelida* belongs to the family Leucisidae and is a benthic, small‐bodied minnow native to the greater Mississippi River basin and is highly adapted for life in large, turbid and free‐flowing rivers. Sturgeon chub distribution, habitat use and associations with other species are well studied in large mainstem rivers, but remain poorly understood in tributaries, which may serve as refugia as mainstem populations decline. Evidence of recent declines in the upper Missouri River highlights the need to better understand the role of these tributary systems. We therefore assessed the distribution and abundance of sturgeon chub populations in western Missouri River tributaries of South Dakota, evaluated broad‐scale abiotic and biotic influences on their distributional patterns and characterized their habitat associations. Sturgeon chub were captured in the Cheyenne (*n* = 81), White (*n* = 331) and Little White (*n* = 71) rivers but were absent from the Little Missouri and Grand rivers. Sturgeon chub distributions were limited to lower areas of all rivers (i.e. closer proximity to mouths) where stream width, turbidity, discharge and observed habitat complexity (i.e. greater diversity of mesohabitats present) were highest. Furthermore, sturgeon chub were often found in association with high abundances (≥10% of catch) of flathead chub *Platygobio gracilis* and *Hybognathus* spp. (plains minnow *Hybognathus placitus* and western silvery minnow *Hybognathus argyritis*). Sturgeon chub generally used main‐ or secondary‐flowing macrohabitats and were predominantly found in channel border or thalweg mesohabitats in most sampled reaches. Mesohabitats with greater bottom velocities [mean = 0.34 m/s, standard deviation (SD) = 0.11] and higher percentages of gravel (mean = 44, SD = 24) in the substrate were more likely to contain sturgeon chub in the White and Little White rivers, whereas greater depths (mean = 0.49 m, SD = 0.19) were associated with their presence in the Cheyenne River. Our study highlights the importance of tributaries as a surrogate to the mainstem Missouri River regarding sturgeon chub persistence that may be applicable to other imperilled fish species.

## 1 INTRODUCTION

Freshwater biodiversity in North America is declining at an alarming rate due to anthropogenic influences, with approximately 40% of freshwater fish documented as imperilled or extinct (Dudgeon, 2019; Jelks et al., 2008; Walsh et al., 2011). Aquatic ecosystems in the Great Plains region of North America are particularly at risk of extinction due to anthropogenic changes that have influenced alterations in flow regime, habitat fragmentation and the spread of introduced species (Dieterman & Galat, 2004; Dodds et al., 2004; Perkin & Gido, 2011). Specifically, modifications to the mainstem Missouri River resulted in an estimated alteration of 1.2 million hectares of river habitat (Dickey, 2011). Thus, these changes in habitat have had negative consequences for native Great Plains river‐obligate species in successfully carrying out their life history (Costigan & Daniels, 2012).

There is a growing body of literature that suggests tributaries may serve as a surrogate to mainstem rivers regarding species conservation and recovery. Some of the relatively large tributaries have a smaller anthropogenic footprint and have been viewed as potential refugia for large‐river specialist species (Dunn et al., 2018; Pracheil et al., 2013). Relatively unaltered tributaries may offer a more complex habitat template and a higher degree of connectivity which are necessary for some large river‐obligate species to successfully carry out their life history (Neely et al., 2009; Pracheil et al., 2009). Small‐bodied fish species may be more susceptible to changes in habitat and have, consequently, an increased risk of extinction (Olden et al., 2007; van der Lee & Koops, 2016). However, despite the higher risk, conservation studies primarily focus on larger, charismatic species (Kopf et al., 2017).

The genus *Macrhybopsis* includes small‐bodied, short‐lived species native to the Great Plains that historically occupied turbid, free‐flowing, unchannelized river reaches with a complex habitat template consisting of sloughs, sandbars and large woody debris (Hesse, 1994). The genus *Macrhybopsis* belongs to a unique guild of pelagic broadcast‐spawning minnows, requiring long lengths of continuous riverine habitat for successful reproduction (Albers, 2014a; Albers, 2014b; Dieterman & Galat, 2004; Hoagstrom et al., 2006; Perkin & Gido, 2011; Starks et al., 2016). Flow alterations, decreased turbidity and loss of connectivity by fragmented habitat are considered the greatest threats to all *Macrhybopsis* species in the Missouri River system (Albers, 2014a; Albers, 2014b; Hesse, 1994; Hesse et al., 1993; NGPC, 2010; Rahel & Thel, 2004; Steffensen et al., 2014; USFWS, 2001a; USFWS, 2001b; USFWS, 2008a; USFWS, 2008b). Altered hydrology and stream fragmentation inhibit the upstream migration of spawning adults and limits downstream drift distances of developing embryos (Perkin & Gido, 2011).

Sicklefin chub *Macrhybopsis meeki* and sturgeon chub *Macrhybopsis gelida* are two *Macrhybopsis* species native to the Missouri and lower Mississippi rivers. A recent study showed that, within the upper Missouri River system, sicklefin chub catch rates were increasing, whereas sturgeon chub rates were decreasing (Braaten et al., 2021). Further, intense monitoring efforts ranging from 1996 to 2018 determined that sturgeon chub were decreasing throughout their range (Wildhaber et al., 2024). Thus, sturgeon chub have experienced extensive population declines and local extirpations and are now occupying only 53% of their historical range (USFWS, 2023), which led to their listing as species of concern in several states within the Missouri River basin. As a benthic species that feeds on aquatic macroinvertebrates and detritus, sturgeon chub serve as a foundational link in the food web, and their declines could have bottom‐up effects by reducing prey availability for the federally endangered pallid sturgeon *Scaphirhynchus albus* (Gerrity et al., 2006; Hesse, 1994).

**FIGURE 1:**
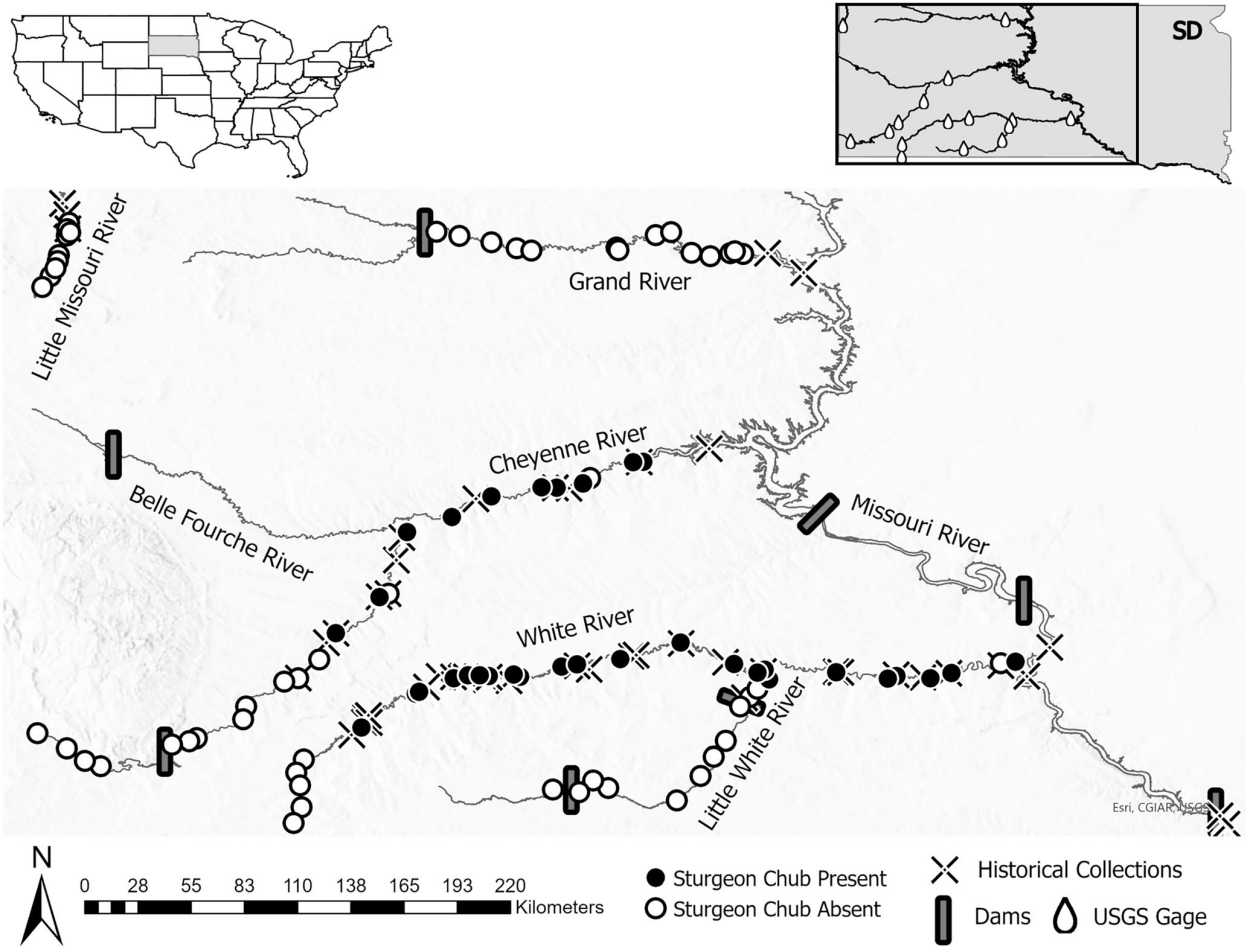
Reaches sampled throughout the study area with sturgeon chub captures are indicated by black circles. Historical sample locations where sturgeon chub were present are also shown. U.S. Geological stations are shown within the inset map of South Dakota.

Sturgeon chub occupancy, habitat use and species associations have been investigated in the mainstem portions of the Missouri and Mississippi rivers (Berry Jr. & Young, 2004; Braaten et al., 2021; Dieterman & Galat, 2004; Everett et al., 2004; Gould, 1994; Grady & Milligan, 1998; Herzog, 2004; Ridenour et al., 2009; Welker, 2000; Welker & Scarnecchia, 2004; Wildhaber et al., 2012), yet limited knowledge exists in their predominant tributaries (Hampton & Berry Jr., 1997; Quist et al., 2004; Stewart, 1981; Werdon, 1992). Viable sturgeon chub populations have been found in some of these tributaries (South Dakota Game, Fish, and Parks, unpublished data) and, as such, their persistence in lotic systems isolated by mainstem reservoirs in the Missouri River suggests that sturgeon chub can fulfil life‐history requirements in relatively smaller systems. However, sturgeon chub have been extirpated from 9 of 21 tributaries the species formerly occupied (USFWS, 2023). In South Dakota alone, monitoring efforts intermittently occurred since 1931 and have yielded low abundances ranging from 0 to 50 per site (South Dakota Game, Fish, and Parks, unpublished data; Figure 1). Thus, understanding their habitat associations in tributaries of South Dakota is imperative regarding species' persistence.

The aim of this study was to better understand the distribution and abundance of sturgeon chub populations in select tributaries of the Missouri River in South Dakota. Our aims were to (1) evaluate broad‐scale abiotic and biotic influences on sturgeon chub distribution patterns, and (2) determine fine‐scale influences of sturgeon chub presence to characterize their habitat use in tributary rivers. These data will help inform conservation and management decisions for sturgeon chub and contribute to the understanding of its persistence in isolated, tributary rivers that may have habitat more reminiscent of the historical Missouri River.

## 2 METHODS

### 2.1 Study area

We sampled five western Missouri River tributaries located in South Dakota that sturgeon chub historically occupied (USFWS, 2023): the Little Missouri, Grand, Cheyenne, White and Little White rivers (Figure 1). The hydrology of these rivers is typical of others in the Great Plains region, where cycling of extreme environmental conditions (i.e. floods, drought) has historically played large roles in shaping fish distribution and assemblage structure (Dodds et al., 2004). Varying degrees of flow regulation are now associated with each of these rivers, except for the White River, which remains undammed (Barth & Sando, 2024). The Little White River meets its confluence with the White River, whereas the remaining rivers have confluences that fall within the most regulated reach of the mainstem Missouri River. Ultimately, mainstem dams and reservoirs limit connectivity between the systems we studied.

Fishes and habitat characteristics were sampled from 90, 1‐km river reaches in the Little Missouri (*n* = 10), Grand (*n* = 14), Cheyenne (*n* = 24), White (*n* = 28) and Little White (*n* = 14) rivers during July–August 2020 and May–August 2021 (Figure 1). The number of reaches were proportionally allocated to each river by its extent (river kilometre) in South Dakota. Reaches were stratified by river and randomly selected from accessible sites (e.g. bridge crossings, adjacent roads or permitted private lands), with priority given to historically sampled locations.

### 2.2 Data collection

Fish community and habitat data collection methods followed standardized procedures developed by the Pallid Sturgeon Population Assessment Team for the Missouri River system (Welker & Drobish, 2016). Specifically, we employed an active sampling approach using three gears (i.e. seine, otter trawl, drifting trammel net) to target sturgeon chub and other benthic fish species within each sample reach. The seine had a width of 7.5 m and a mesh‐size of 10 mm, and the otter trawl had a width of 2.4 m and a mesh‐size of 5 mm. The 30‐m multifilament nylon trammel net had a 25‐mm inner mesh and a 200‐mm outer mesh and was utilized to capture large‐bodied species.

We used a two‐tiered hierarchical habitat classification system, designating each net sample with a macrohabitat and a mesohabitat type (Welker & Drobish, 2016). In particular, we used macrohabitat data for descriptive habitat associations at a broad scale and then mesohabitat data to determine sturgeon chub presence at a fine scale. Macrohabitat types included main channel, off‐channel (e.g. connected side channels, braided channels) and backwater (e.g. disconnected channels). Mesohabitat types included bar (shallow depositional areas), pool (deep, low‐flow habitats), riffle–run (fast, shallow habitats) and thalweg/border (deep channel or channel‐edge habitats). We matched our sampling approach to stream conditions in an attempt to encompass all available macro‐ and mesohabitat types within each reach and prioritized seine and trawl deployment to best capture sturgeon chub presence and absence among net samples and reaches, because the mesh‐size of the trammel net limited captures to large‐bodied species. Time, Global Positioning System (GPS) coordinates, reach length (1‐km goal) and number of total net samples (six sample minimum) were recorded for each reach.

We deployed seines, trawls and drifting trammel nets in a downstream direction for the full length of the mesohabitat within a given reach or up to 100 m during daylight hours. Half arc and quarter arc methods were also used with the seine to sample a known area (Welker & Drobish, 2016). All fishes captured were identified and enumerated. Total length, measured to the nearest millimetre, for all fish species was recorded. Fish were released near their capture location after processing.

We collected physical measures of habitat at the midpoint of all net sample locations. Depth (m) was measured using a standard United States Geological Survey (USGS) top setting wading rod. The top setting wading rod was paired with a Marsh McBirney Flo‐Mate 2000 to measure velocity (m/s). When water depth exceeded 1.2 m, velocity measurements were taken at the river bottom, at 0.2 m of depth and at 0.8 m of depth. Velocity measurements were taken at the bottom and at 0.6 m of water depth when depth was <1.2 m. We sampled substrate using a hand‐held shovel and visually estimated sediment types after flushing water over the sample and through 2‐ and 0.630‐mm sieves. Cobble and organic matter were categorized as none, incidental, dominant or ubiquitous. The percentages of gravel, sand, silt and compacted sediment were visually estimated and summed to 100%, unless cobble was ubiquitous. Water temperature (°C) and dissolved oxygen (mg/L) were measured using a YSI‐85 instrument. A Lovibond TB 250 WL portable turbidimeter was used to measure turbidity in nephelometric turbidity units (NTU), and conductivity (μS) was measured using a Hach conductivity meter. River stage and discharge data were determined by the nearest USGS gage post‐sampling (see Figure 1, inset map). Mean wetted stream width (m) for each reach was estimated post‐sampling using 2020 satellite imagery in Google Earth Pro. For each reach, three evenly spaced width measurements were taken, and their average was used to represent mean wetted width.

### 2.3 Data analysis

#### 2.3.1 Relative abundance

We calculated percentage composition (percentage of total catch) for sturgeon chub for each river using all fishes captured within reaches to allow comparison with previous assessments. Additionally, catch per unit of effort (CPUE) was calculated for each gear that captured sturgeon chub within each reach, expressed as the number of individuals per 100 m^2^. Sampling effort for each event was quantified in linear square metres by calculating the net sample area as the product of sampling distance and net width; trawls had a fixed net width, whereas seine widths were adjusted based on mesohabitat conditions. The area of the net was calculated for half arc (1/2πr^2^) and quarter arc (1/4πr^2^) seine methods by using the width between sampling personnel as the radius. Because this was the first study to use trawling techniques in the study area, direct comparisons with prior abundance estimates were not possible; CPUE values are reported for future reference when these gears are used again.

#### 2.3.2 Multi‐scale analyses

We aimed to understand what factors influenced sturgeon chub presence on two spatial scales: reach scale and mesoscale. First, we conducted analyses to evaluate what abiotic and biotic factors influenced sturgeon chub presence to characterize their distributional patterns at the reach scale. Second, we used data collected by each net sample to characterize patterns at the mesoscale as net samples were delineated by mesohabitat type.

#### 2.3.3 Reach‐scale distributional patterns

We used a random forest (Breiman, 2001) classification model to identify abiotic and biotic associations of sturgeon chub reach presence. Random forest is a machine learning algorithm based on regression or classification trees that has been successfully applied to distributional studies of freshwater fish (Vezza et al., 2015). Random forest grows many classification or regression trees based on bootstrap samples of a dataset and combines the predictions from all the trees (Cutler et al., 2007; Murphy et al., 2010). Trees are fit to each bootstrap sample, which contains about two thirds of the original dataset. The remaining one third of the data are referred to as out‐of‐bag (OOB) observations. The OOB error estimates are essentially cross‐validated accuracy estimates, because OOB observations were not used in the fitting of the trees (Cutler et al., 2007).

Net sample habitat and fish data were used to create reach‐scale explanatory variables for splits in the random forest classification model. Measured habitat variables were averaged by reach and mesohabitat type to provide a composite description of habitat in the entire reach. These averages were calculated to give equal weight to each mesohabitat type and account for sample differences among reaches. Continuous habitat variables were z‐score standardized prior to analysis to account for differences in measurement units and value ranges of environmental data. Fish community abundance variables were created for species that we hypothesized to be ecologically relevant to sturgeon chub presence or absence (i.e. niche overlap, competitors, predators) by transforming the summed reach data for each species into categorized percentage of catch variables [0: absent (abs), 1: <10% of catch (pres), 2: ≥10% of catch (abu); Vezza et al., 2015]. Fish species that have been considered ecologically relevant to sturgeon chub presence or absence include flathead chub *Platygobio gracilis* and *Hybognathus* species due to similar life histories (Gould, 1994; Grisak, 1996; Quist et al., 2004; Stewart, 1981; Welker & Scarnecchia, 2004; Werdon, 1992; Wildhaber et al., 2012); sauger *Sander canadensis*, channel catfish *Ictalurus punctatus* and *Micropterus* species due to predation (Fryda, 2001; Gardner & Berg, 1982; Hoagstrom et al., 2007; Rahel & Thel, 2004; Weitzel, 2002); red shiner *Cyprinella lutrensis* and sand shiner *Notropis stramineus* due to common occurrence within the same systems (Quist et al., 2004); and longnose dace *Rhinichthys cataractae* due to niche overlap (Stewart, 1981).

Generalized variance inflation factor (GVIF) scores were used to assess multicollinearity of explanatory variables. Specifically, we used GVIF^1/2(df)^, where df represents degrees of freedom, to allow comparison between the different dimensions or varying degrees of freedom of continuous and categorical variables. Highly collinear variables were sequentially excluded from the analysis based on their hypothesized ecological importance to sturgeon chub occupancy, until all GVIF^1/2(df)^ scores were ≤3 (Fox & Monette, 1992; Zuur et al., 2010). However, random forest averages over multiple trees and randomly selects variables for each split, allowing the influence of groups of correlated variables to be distributed over the forest. Therefore, the random forest algorithm can comprehensively explore the predictor space and thus model complex interactions between variables even if collinearity exists (Cutler et al., 2007).

The random forest classification model included 19 explanatory variables (Table 1) and a 1 binary response variable: sturgeon chub presence (=1) or absence (=0). We calculated the mean importance values for each variable from 10 runs of random forest to address the randomness associated with the machine learning algorithm. For each run of the model, we built 10,000 trees, with two variables tried at each split, using default bootstrap resampling and no tree discards. Model stability was assessed by examining the OOB error, which plateaued before 2000 trees, ensuring sufficient iterations. The model improvement ratio (Murphy et al., 2010) technique was employed to identify the most parsimonious model. Partial dependence plots were constructed to visualize the effect of each covariate included in the selected model. Relative variable importance (IMP) and model performance metrics, including OOB error, accuracy, sensitivity, specificity, Cohen's kappa (*k*), area under the receiver operating characteristic curve (AUC) and true skill statistic (TSS), were used to evaluate the model fit to the data (Mouton et al., 2010; Vezza et al., 2015). All random forest model fitting, selection and evaluation were performed using the randomForest (Liaw & Wiener, 2002) and rfUtilities (Evans & Murphy, 2018) packages in programme R, version 4.1.3 (R Core Team, 2021).

**TABLE 1:** Abiotic and biotic variables included in reach‐scale analyses with units of measurement and ranges.

Variable	Unit	Range
Abiotic		
Reach distance	m	442–1400
Number of samples	n	6–13
Stream width	m	4–86
Discharge	cfs	8–507
Temperature	°C	18–31
Turbidity	NTU	21–1100
Conductivity	μS/cm	269–2465
Dissolved oxygen	mg/L	4–12
Depth^a^	m	0.16–0.71
Bottom velocity^a^	m/s	0.05–0.36
Sand	%	8–96
Gravel	%	3–63
Silt^a^	%	1–65
Compacted^a^	%	0–28
Cobble	0/1/2/3	0–3
Organic	0/1/2/3	0–1
Biotic		
Flathead chub	0/1/2	0–2
*Hybognathus* spp.	0/1/2	0–2
Longnose dace	0/1/2	0–2
Red shiner	0/1/2	0–2
Channel catfish	0/1/2	1–2
Sauger	0/1/2	0–1
*Micropterus* spp.	0/1/2	0–2

*Note*: Abiotic variables were used in both random forest and nonmetric multidimensional scaling (NMDS) procedures, except when noted below. Categorical fish community variables (0: absent, 1: present, 2: abundant or >10% of catch) were used in random forest procedures, whereas a matrix of fish community presence and absence were used as the response for the NMDS. Reach distance and number of net samples were included to account for different levels of effort among reaches. Categorical levels for cobble and organic were as follows; 0: none, 1: incidental, 2: dominant and 3: ubiquitous.

^a^ Variables are excluded from random forest procedures due to multicollinearity.

Abbreviation: NTU, nephelometric turbidity unit.

We also aimed to better understand the biotic associations of sturgeon chub by investigating the differences in total fish community composition among reaches in relation to sturgeon chub presence. Permutational multivariate analysis of variance (PERMANOVA) was utilized to test for differences in fish community composition among sturgeon chub present and absent reaches. If significant differences were found, fish community relationships were then visualized using nonmetric multidimensional scaling (NMDS), an ordination technique that is widely used to depict fish community patterns (Beard et al., 2018; Helms et al., 2005; Ruoss et al., 2023; Smith et al., 2016). NMDS reduces dimensionality and arranges points within the ordination space so that the rank order of dissimilarities is maintained (Giddings et al., 2006). The fit of the ordination was evaluated by the stress value, with a value ≤0.20 indicative of a good fit (Beard et al., 2018; McCune & Grace, 2002). Jaccard distance was used for the NMDS ordination performed using R package vegan (Oksanen et al., 2022). Reach‐specific presence–absence data were used to form the ordination. Sturgeon chub were removed from the community matrix prior to the ordination procedure to exemplify differences in species compositions independent of sturgeon chub presence. Sturgeon chub presence was then added as a categorical grouping variable to visualize and assess the differences in fish communities between reaches where they were present or absent. Confidence ellipsoids of sturgeon chub present and absent reaches were constructed with an 80% confidence interval of the standard deviation (SD) and projected onto the ordination.

Finally, multiple regression analysis of z‐score‐scaled habitat variables (Table 1) and ordination axes was conducted using the vegan package (function envfit; Oksanen et al., 2022) to identify which abiotic variables were driving the differences in fish community composition if such relationship was detectable. A Bonferroni correction was used to account for the multiple comparisons (Beard et al., 2018) where a corrected value of *p* < 0.005 was used to determine the significance of abiotic variables to the ordination. Significant variables were then projected as vectors onto the ordination space.

#### 2.3.4 Mesoscale presence

We fit multiple binomial logistic regression models to better characterize sturgeon chub mesohabitat use by investigating the influences of environmental variables on sturgeon chub presence within net samples. Reaches where sturgeon chub were not captured were removed from these analyses to reduce noise from observations collected outside of sturgeon chub–occupied areas. The response variable (i.e. sturgeon chub presence/absence) and environmental explanatory variables (Table 1) were selected for evaluation if they were hypothesized as being influential to sturgeon chub presence based on previous literature and were deemed appropriate for analyses at this scale (i.e. varied substantially between mesohabitat samples). The White River model set included data from both the White River and Little White River net samples due to the connectivity of these systems and the limited differences in selected habitat variables.

Explanatory variables were z‐score scaled and evaluated for multicollinearity with a GVIF cut‐off of 3 (Fox & Monette, 1992; Zuur et al., 2010). Separate models were developed for each river drainage to account for differences in measured habitat variables between rivers. A model list was prepared for each analysis using all possible combinations of explanatory variables to predict sturgeon chub presence at the mesohabitat scale. Corrected Akaike information criterion (AICc) for small sample size model selection was used to determine the most optimal combination of covariates. Models holding approximately 95% of the cumulative weight within the list were averaged to obtain more accurate coefficients for each term (Burnham & Anderson, 2002). Fully averaged coefficients were used to construct prediction plots of sturgeon chub probabilistic mesohabitat presence for the most strongly related terms by holding all other terms in the averaged model to their mean.

## 3 RESULTS

A total of 483 sturgeon chub were captured across study sites, with 331 in the White River, 71 in the Little White River and 81 in the Cheyenne River (Figure 1). Sturgeon chub were captured in 22 of the 28 (79%) White River reaches, 10 of the 24 (42%) Cheyenne River reaches and 3 of the 14 (21%) Little White River reaches. No sturgeon chub were captured in the Grand or Little Missouri River. Percentage composition of sturgeon chub was highest in the White River (mean = 7.4%, SD = 7.3%), followed by the Cheyenne River (mean = 1.9%, SD = 4.2%) and the Little White River (mean = 1.1%, SD = 2.4%). The trawl captured 82% of individuals, and the seine captured 18%. Trawl CPUE was highest in the White River, followed by the Little White and Cheyenne rivers, whereas seine CPUE was low overall, and no sturgeon chub were captured using seine in the Little White River (Table 2).

**TABLE 2:** Means and standard deviations of CPUE (fish per 100 m^2^) by sampling method and river.

Sampling method	Cheyenne River	White River	Little White River
Trawl CPUE	0.27 (0.52)	1.32 (1.36)	0.72 (1.49)
Seine CPUE	0.02 (0.05)	0.29 (0.42)	0

*Note*: Standard deviations are shown in parentheses.

Abbreviation: CPUE, catch per unit of effort.

The White River exhibited the largest geographical distribution of sturgeon chub where they were generally captured throughout the entirety of the tributary except for reaches within 309 river kilometres of the headwaters (Figure 1). Comparatively, we captured only sturgeon chub in the lower 7 river kilometres of the Little White River, which was the smallest extent within a tributary we observed. Sturgeon chub were absent from reaches upstream and within 136 river kilometres downstream of Angostura Dam in the Cheyenne River. Particularly, sturgeon chub abundances in the Cheyenne River were highest between the confluence of the Belle Fourche River and the river mouth (Figure 1), where 86% of the total catch was recorded.

**FIGURE 2:**
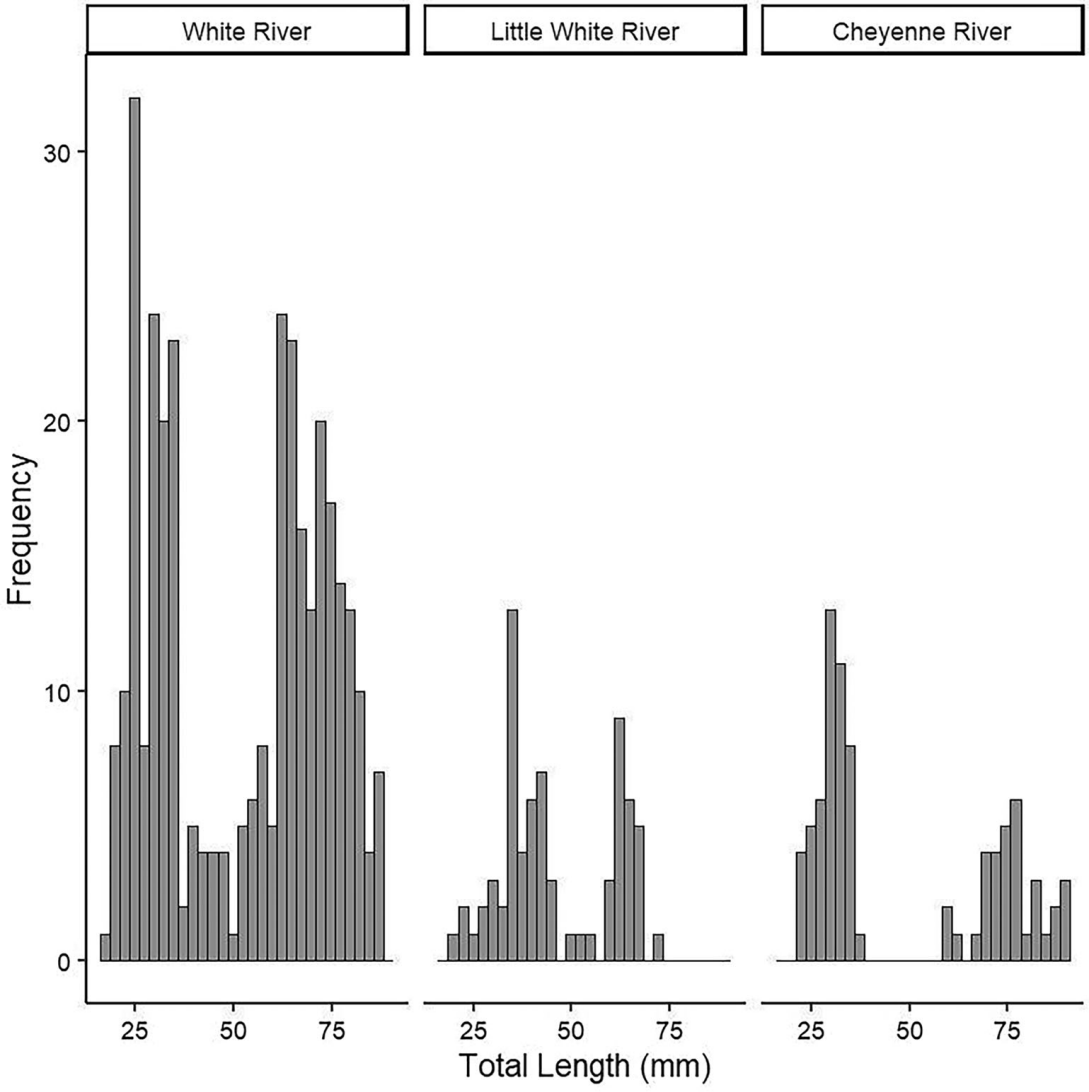
Length–frequency histograms showing individual lengths of sturgeon chub captured from the White (*n* = 331), Little White (*n* = 71) and Cheyenne (*n* = 81) rivers [minimum = 18 mm, maximum = 90 mm, mean = 51 mm, SD (standard deviation) = 21 for all rivers].

**FIGURE 3:**
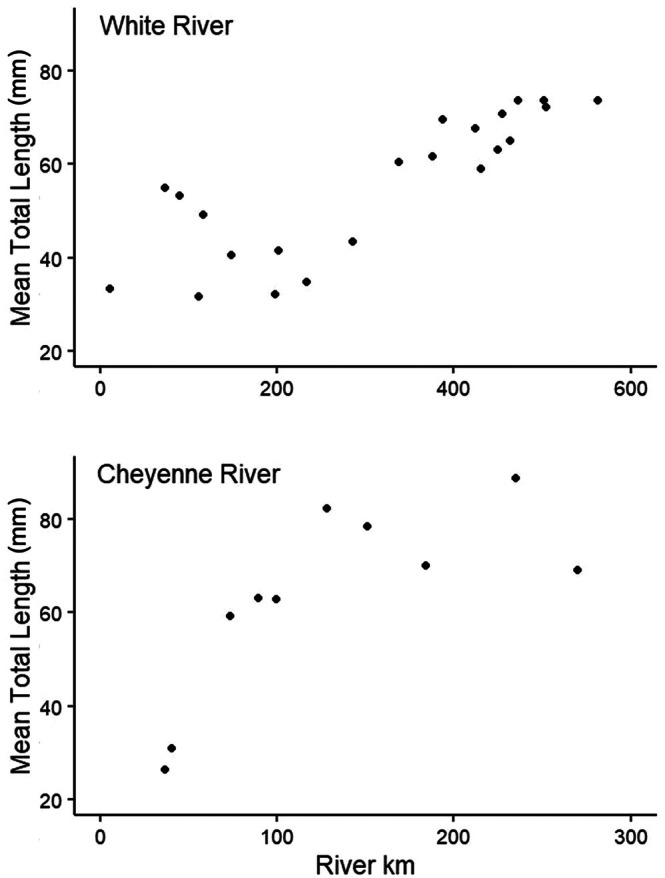
Sturgeon chub mean total length for each reach where they were captured plotted against river kilometre on the White and Cheyenne rivers. Zero on the *x*‐axis represents the river mouth.

Sturgeon chub total length ranged from 18 to 90 mm (mean = 51, SD = 21; Figure 2). Length–frequency distributions differed by river system, with the White River having the most even distribution of individuals among all length classes. We observed that distribution of size structure for sturgeon chub also varied along the river gradient in the Cheyenne and White rivers. Generally, larger sturgeon chub were captured in the uppermost reaches of their occupied areas, whereas smaller, presumably age‐0 fish were predominantly located in downstream reaches (Figure 3). The lower Little White River generally had a combination of sturgeon chub size structure throughout their occupied range. Observations of larger individuals in the upper reaches indicated spawning activity, including aggregations of males and females, gravid females and males with expressed milt.

**FIGURE 4:**
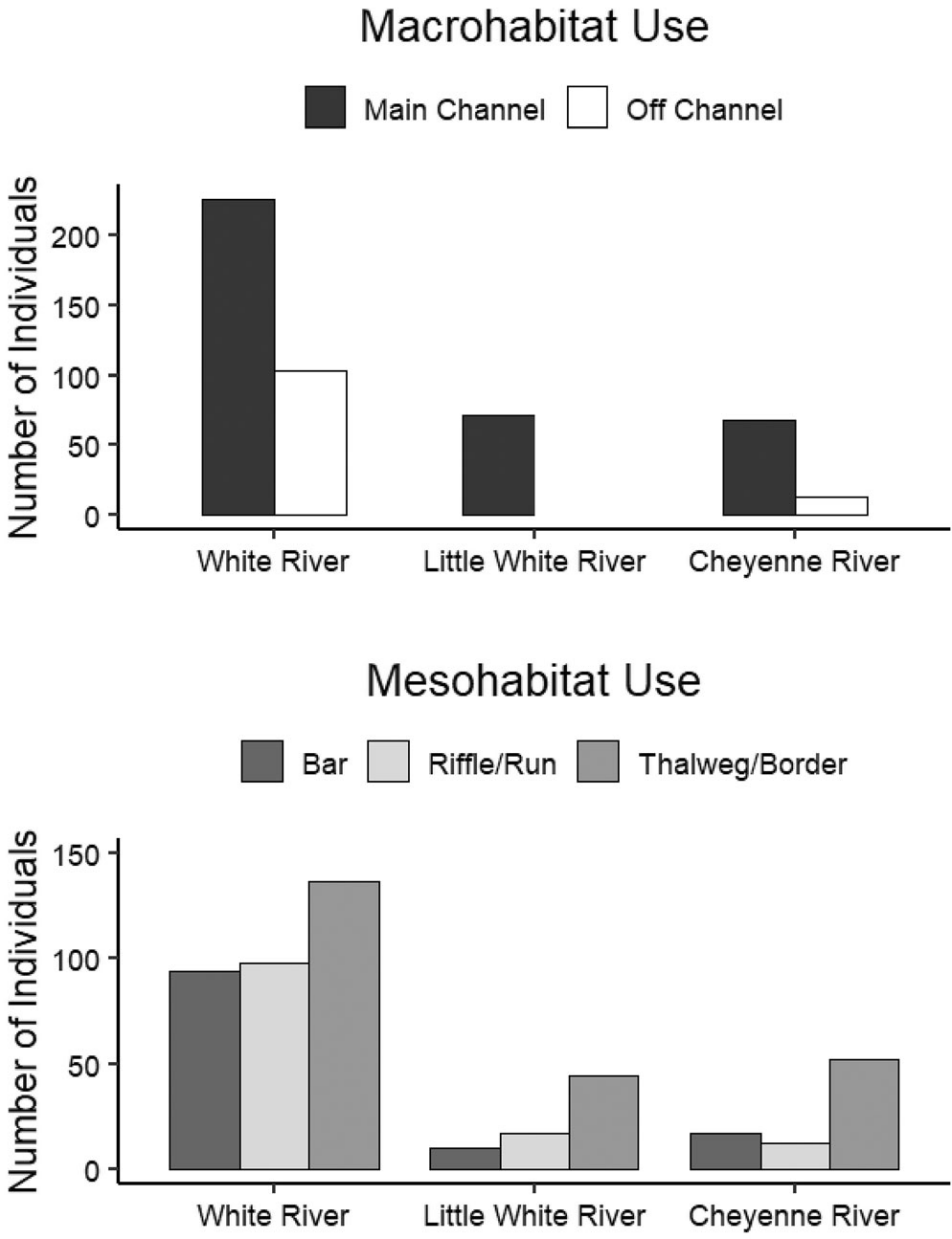
Macrohabitat and mesohabitat associations by sturgeon chub in each river that they were captured in. The number of individuals represents the cumulative sum of sturgeon chub captured with all sampling gear used. No sturgeon chub were captured in off‐channel habitats in the Little White River. No captures of sturgeon chub occurred from backwater macrohabitats or pool mesohabitats.

We captured 76% of all sturgeon chub in main channel macrohabitats and 24% in off‐channel macrohabitats. No sturgeon chub were captured in backwater macrohabitats. Sturgeon chub mesohabitat association consisted of 25% bars, 27% riffle–runs and 48% thalweg/borders. We did not collect sturgeon chub in pool mesohabitats. Sturgeon chub macro‐ and mesohabitat use varied between rivers (Figure 4), with fish from the White River utilizing a greater diversity of habitat classes. Sturgeon chub associations with measured variables also varied among rivers (Table 3); however, they were most frequently associated with bottom velocities of 0.32–0.34 m/s and depths of 0.25–0.49 m over substrates of gravel and sand.

**TABLE 3:** Means, standard deviations and ranges of measured habitat variables of sturgeon chub present net samples from the Cheyenne, White and Little White rivers.

Variables	Cheyenne River	White River	Little White River
Stream width (m)	38 (22)	43 (23)	20 (18)
Range	9–86	7–83	4–59
Mean discharge (cfs)	267 (50)	156 (113)	71 (0)
Range	207–394	11–408	71–71
Mean temperature (°C)	25.81 (1.83)	26.14 (3.14)	28.96 (2.48)
Range	18.90–29.10	18.20–31.90	23.50–31.20
Mean turbidity (NTU)	267 (189)	840 (320)	92 (13)
Range	24–800	167–1100	76–111
Mean conductivity (μS/cm)	1994 (123)	632 (74)	348 (21)
Range	1390–2250	416–900	328–394
Mean dissolved oxygen (mg/L)	8.56 (0.38)	8.27 (0.64)	9.03 (0.38)
Range	8.23–11.24	6.79–9.38	8.54–9.70
Mean depth (m)	0.49 (0.19)	0.28 (0.18)	0.25 (0.08)
Range	0.16–0.92	0.06–0.80	0.14–0.48
Mean bottom velocity (m/s)	0.32 (0.10)	0.33 (0.13)	0.34 (0.09)
Range	0.15–0.62	0.10–0.74	0.19–0.59
Substrate			
Mean silt (%)	0 (1)	1 (2)	2 (2)
Range	0–5	0–20	0–5
Mean sand (%)	40 (23)	43 (26)	56 (23)
Range	0–95	0–99	25–95
Mean gravel (%)	55 (25)	47 (27)	41 (21)
Range	0–90	0–95	5–75
Mean compacted (%)	5 (22)	7 (23)	2 (4)
Range	0–100	0–100	0–10
Mode cobble	1 (incidental)	0 (none)	0 (none)
Range	0–2	0–3	0–1
Mode organic	0 (none)	0 (none)	1 (incidental)
Range	0–1	0–1	0–1

*Note*: Standard deviation are shown in parentheses.

Abbreviation: NTU, nephelometric turbidity unit.

### 3.1 Reach‐scale distributional patterns

**FIGURE 5:**
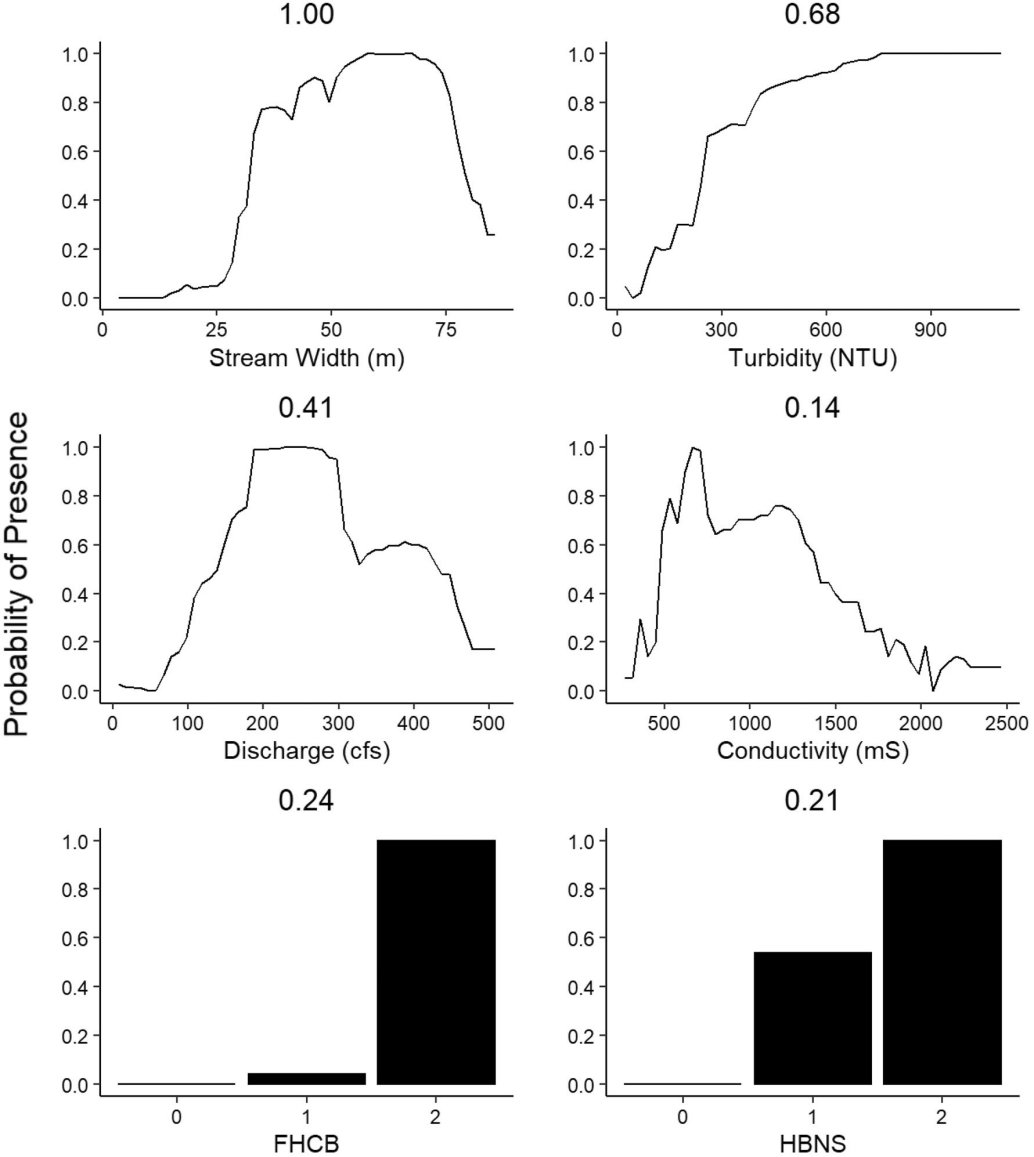
Partial dependence plots with scaled probability of sturgeon chub presence (*y*‐axis) for each variable included in the final random forest model at the reach scale. Abiotic variables were continuous, and biotic variables (FHCB: flathead chub, HBNS: *Hybognathus* spp.) represented categorical abundances (0: absent, 1: <10% of catch, 2: >10% of catch). Relative variable importance is shown above each plot.

Performance metrics of our random forest model suggest high predictive power (OOB error = 6.06%, accuracy = 93.94%, sensitivity = 0.97, specificity = 0.92, *k* = 0.88, AUC = 0.94, TSS = 0.87). Habitat variables of stream width (IMP = 1.00), turbidity (IMP = 0.68), discharge (IMP = 0.41), conductivity (IMP = 0.14) and fish community variables (abs/pres/abu) of flathead chub (IMP = 0.24) and *Hybognathus* spp. (plains minnow *H. placitus* and western silvery minnow *Hybognathus argyritis*; IMP = 0.21) were retained for the final random forest model. Partial dependence plots reveal that turbidity has a consistently positive relationship with sturgeon chub presence in a reach (Figure 5). The probability of sturgeon chub presence is highest within optimal ranges of stream width (30–75 m), discharge (150–450 cfs) and conductivity (500–1500 μS/cm). The abundances of flathead chub and *Hybognathus* spp. were both positively correlated with the probability of sturgeon chub presence (Figure 5).

**FIGURE 6:**
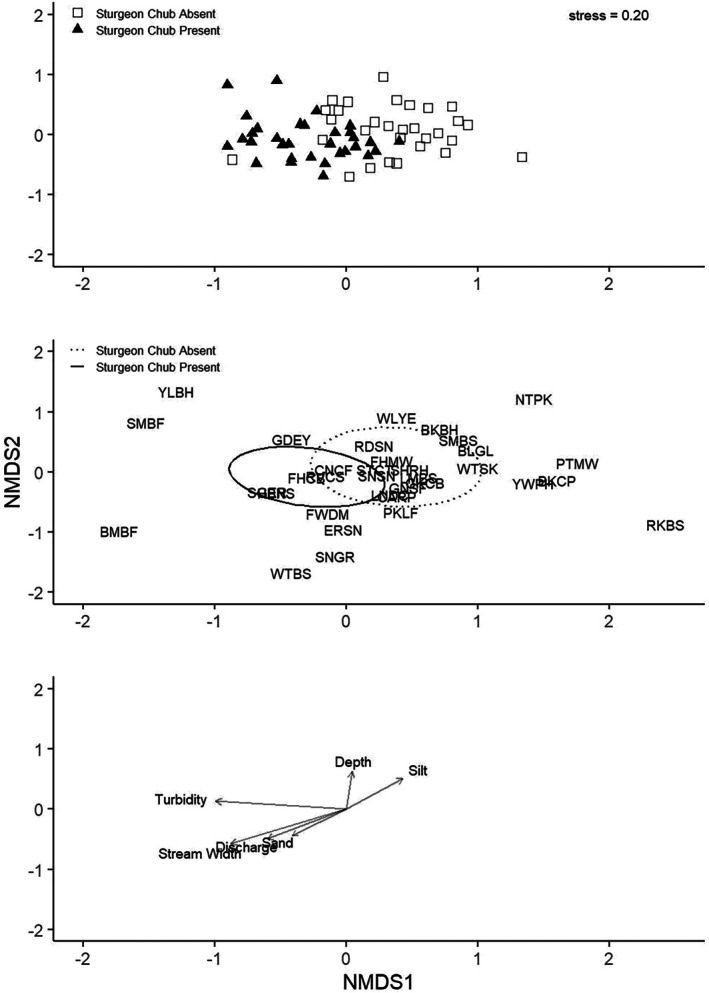
Nonmetric multidimensional scaling (NMDS) using Jaccard distance formed with species presence and absence from White, Little White and Cheyenne river reaches. Sturgeon chub were removed from the matrix and included as a categorical grouping variable to evaluate species composition differences between sturgeon chub present and absent reaches. The upper plot is the reach ordination with sturgeon chub present and absent reach indicated and contains the stress value on two dimensions. The middle plot is the species ordination (see Table S1 for species codes) with the 80% standard deviational confidence ellipsoids of the sturgeon chub present and absent groups. The lower plot shows significant abiotic variables to the ordination as vectors.

The PERMANOVA indicated fish community composition differed based on sturgeon chub presence (*p* < 0.001, psuedo‐*R* ^2^ = 0.13). Moreover, differences in community composition among rivers was important and potentially confounding (*p* < 0.001, psuedo‐*R* ^2^ = 0.15). The interaction of these two variables (*p* < 0.001, psuedo‐*R* ^2^ = 0.08) indicates the rivers possess distinct fish communities, with some overlap in community composition occurring in reaches that contain sturgeon chub. The NMDS ordination (stress = 0.20) indicates sauger and *Hybognathus* spp. were most frequently associated with the presence of sturgeon chub, whereas red shiner, plains killifish *Fundulus zebrinus*, shorthead redhorse *Moxostoma macrolepidotum*, white sucker *Catostomus commersonii*, creek chub *Semotilus atromaculatus*, black bullhead *Ameiurus melas*, walleye *Sander vitreus*, bluegill *Lepomis macrochirus*, largemouth bass *Micropterus nigricans* and smallmouth bass *Micropterus dolomieu* were frequently associated with their absence (Figure 6).

Stream width, turbidity, discharge, percent sand, depth and percent silt were related to the species occurrence ordination (Bonferroni‐corrected *α* value of *p* < 0.005) and, consequently, differences in fish community composition. Stream width, discharge and percent sand were highest in reaches containing sturgeon chub, whereas the most turbid reaches were associated with sturgeon chub in occupied areas of the White River. Increasing depth and silt related to the absence of sturgeon chub (Figure 6).

### 3.2 Mesohabitat‐scale presence

**FIGURE 7:**
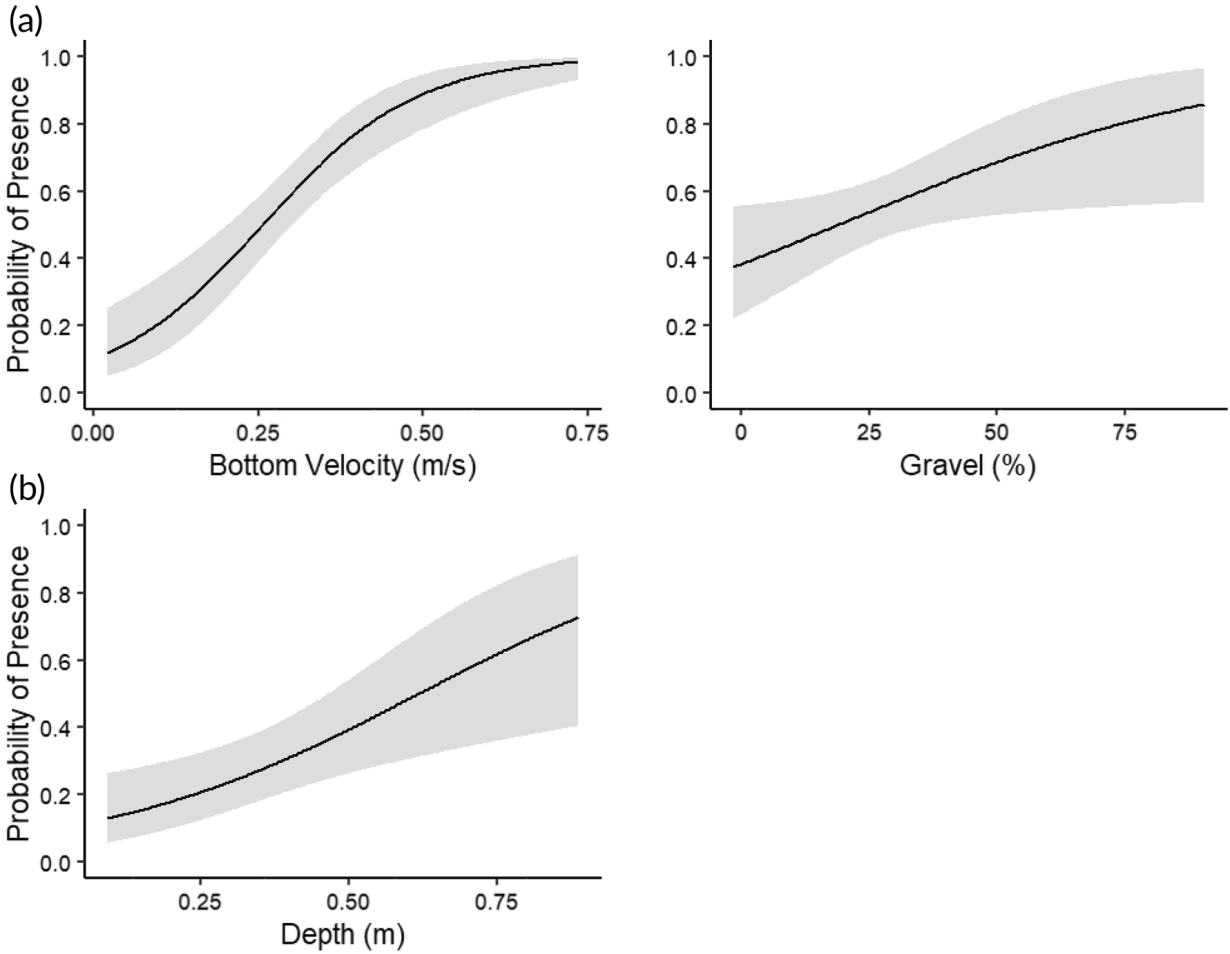
Variables found to have the strongest relationship with sturgeon chub presence at the mesohabitat scale from logistic regression analyses of the (a) White and Little White rivers and (b) Cheyenne River. The probability of sturgeon chub presence in a mesohabitat was predicted for each variable by holding all over variables included in the model to their means. The shaded regions represent the 95% confidence intervals of the predictions.

The model with the lowest AICc score for the White and Little White rivers included bottom velocity and gravel; however, percent silt and percent compacted substrate were removed from the model selection process due to multicollinearity. This model held 40% of the cumulative weight of the 15 models included in the list (Table 4). The model averages of coefficients for covariates of bottom velocity and gravel exhibited strong positive relationships with the probability of sturgeon chub presence at the mesohabitat scale for the White and Little White rivers (Figure 7a).

**TABLE 4:** AICc logistic regression model selection table for the White River system (White River and Little White River).

Candidate model	AICc	*k*	ΔAIC	Weights	cumw
Velocity + gravel	195.35	4	0.00	0.42	0.42
Velocity + gravel + depth	196.43	5	1.08	0.24	0.66
Velocity + gravel + sand	196.73	5	1.38	0.21	0.87
Velocity + gravel + depth + sand	197.94	6	2.59	0.11	0.98
Velocity + sand	203.14	4	7.79	0.01	0.99
Velocity	204.25	3	8.91	0.00	0.99
Velocity + depth + sand	205.08	5	9.73	0.00	1.00
Velocity + depth	206.16	4	10.81	0.00	1.00
Depth + gravel	225.15	4	29.80	0.00	1.00
Depth + gravel + sand	227.17	5	31.82	0.00	1.00
Gravel	228.33	3	32.99	0.00	1.00
Depth	230.24	3	34.89	0.00	1.00
Depth + sand	251.73	4	56.39	0.00	1.00
Gravel + sand	252.13	4	56.79	0.00	1.00
Sand	252.14	3	56.79	0.00	1.00

*Note*: The top four models were model averaged. *k* is the number of parameters, ΔAICc refers to delta AICc, *weight* pertains to the weight of each model and *cumw* refers to the cumulative weight of the model.

Abbreviation: AICc, corrected Akaike information criterion.

Variables of depth and gravel were included in the top model for the Cheyenne River, which held 27% of the cumulative weight of the 15 models included in the list (Table 5). The full average of the estimated coefficient of depth exhibited the strongest relationship with sturgeon chub, with presence increasing with depth (Figure 7b).

**TABLE 5:** AICc logistic regression model selection table for the Cheyenne River.

Candidate model	AICc	*k*	ΔAICc	Weights	cumw
Depth + gravel	104.65	4	0.00	0.27	0.27
Velocity + gravel + depth	105.02	5	0.37	0.22	0.49
Depth + gravel +sand	106.13	5	1.48	0.13	0.62
Velocity + gravel + depth + sand	107.02	6	2.37	0.08	0.78
Velocity + depth	107.27	4	2.62	0.07	0.69
Depth	107.61	3	2.96	0.06	0.84
Velocity + gravel	108.37	4	3.72	0.04	0.88
Velocity + depth + sand	109.21	5	4.56	0.03	0.91
Velocity	109.30	3	4.65	0.03	0.93
Depth + sand	109.55	4	4.90	0.02	0.96
Velocity + gravel + sand	110.62	5	5.97	0.01	0.97
Gravel	110.93	3	6.28	0.01	0.98
Velocity + sand	111.40	4	6.75	0.01	0.99
Gravel + sand	112.23	4	7.58	0.01	1.00
Sand	114.34	3	9.69	0.00	1.00

*Note*: The top nine models were model averaged. *k* is the number of parameters, ΔAICc refers to delta AICc, *weight* pertains to the weight of each model and *cumw* refers to the cumulative weight of the model.

Abbreviation: AICc, corrected Akaike information criterion.

## 4 DISCUSSION

We captured sturgeon chub in three of the five tributaries sampled: the White, Little White and Cheyenne rivers. Our model results at the reach scale suggested that sturgeon chub presence was associated with stream width, turbidity, discharge and conductivity as well as higher abundances of flathead chub and other *Hybognathus* species. Interestingly, turbidity was the only abiotic variable at the reach scale that continually had a positive relationship with sturgeon chub presence. At the mesoscale, our model results showed that sturgeon chub presence was most strongly associated with greater bottom velocity and gravel substrate in the White and Little White rivers. However, sturgeon chub presence was strongly associated with greater depth in the Cheyenne River.

We did not capture sturgeon chub in the Grand and Little Missouri River tributaries. This was not surprising as the last records of sturgeon chub in these rivers were in the 1960s and 1970s (SDGFP, n.d.; Bich & Scalet, 1977). Extended drought (Kelsch, 1994) and damming (Bailey & Allum, 1962) have been attributed to the extirpation of sturgeon chub in these tributaries despite efforts to re‐establish them specifically in the Little Missouri River between 1998 and 2000 (Rahel & Thel, 2004; USFWS, 2001b).

We observed the highest catch of sturgeon chub from the White, Little White and Cheyenne rivers ever recorded (Fryda, 2001; Jones, 2018). Historical records starting in 1931 demonstrated low captures of sturgeon chub throughout the state, ranging from 0 to 50 per sampling event. The river that yielded 50 sturgeon chub at one site was the White River, though this occurred in 1998, and similar counts per site have not been recorded since (South Dakota Game, Fish, and Parks, unpublished data). Other studies documenting sturgeon chub composition in these tributaries reported a composition between 1% and 4% (Fryda, 2001; Jones, 2018), whereas we found sturgeon chub comprised between 2% and 7%. Particularly, the White River also yielded the highest number of sturgeon chub compared to the Little White and Cheyenne rivers for our study. Populations of sturgeon chub are likely to fluctuate over time, and the reproductive success and abundances are reflective of streamflow conditions, similar to other pelagic spawning minnows in the Great Plains (Durham & Wilde, 2006; Durham & Wilde, 2009; Wilde & Durham, 2008; Worthington et al., 2014). The flow regimes of the Cheyenne, White and Little White rivers are relatively unaltered and often experience periods of low or high discharge due to stochastic environmental conditions (Hoagstrom, 2006). Therefore, it is plausible that the extremely high‐water years of 2018 and 2019 aided the reproductive success of sturgeon chub populations in these rivers and contributed to the greater abundances we recorded during our study. These increased captures of sturgeon chub during our study could indicate a recent trend in population growth attributed to high discharges, though we also acknowledge it could be due to differing capture efficiency between benthic trawls (this study) and backpack electrofishing and seines (historical records, e.g. Cunningham & Hickey, 1997). Specifically, our study was the first to use trawling techniques to target benthic‐oriented fishes in these systems, and trawls can be more effective in capturing sturgeon chubs and associated species. For example, sicklefin chub and sturgeon chub captured in the mainstem Missouri and Mississippi rivers using trawling techniques showed both species to be more common than previously thought, resulting in the denial of their listing to the Endangered Species Act in 2001 (Grady & Milligan, 1998; Grisak, 1996; USFWS, 2001).

The spatial distribution along river gradients we observed may provide evidence of seasonal movements or dispersals that have not been previously identified for the species. We recorded larger, actively spawning sturgeon chub in farther upstream reaches of the White and Cheyenne rivers compared to smaller and young‐of‐the‐year individuals in the downstream reaches. These observations could be indicative of movements associated with spawning as observed in other *Macrhybopsis* species such as the peppered chub *Macrhybopsis tetranema*, where larger, sexually mature fish move upstream to spawn, whereas smaller juveniles remain downstream (Bonner, 2000). Although sturgeon chub were captured along a longitudinal gradient, their absence at historically occupied upstream sites may be considered concerning. The lack of persistence in the uppermost reaches of these tributaries may be attributed to factors such as variable streamflow (i.e. lack of longitudinal connectivity), decreased turbidity and narrower stream width. Particularly, these variables increase as proximity to the confluence with the Missouri River increases, which may explain why sturgeon chub has persisted in the downstream reaches. Our model results also corroborated increasing discharge, turbidity and stream width, within certain thresholds, increase the probability of sturgeon chub presence. Our study is one of few to describe broad‐scale and finer‐habitat associations for sturgeon chub in relatively small rivers (Hampton & Berry Jr., 1997; Quist et al., 2004; Stewart, 1981; Werdon, 1992). Comparatively, in large, mainstem rivers, sturgeon chub have been documented in habitats with sustained flow and not in backwater habitats (Berry Jr. & Young, 2004; Braaten et al., 2021; Everett et al., 2004; Gould, 1994; Grady & Milligan, 1998; Herzog, 2004; Ridenour et al., 2009; Welker, 2000; Welker & Scarnecchia, 2004; Wildhaber et al., 2012), similar to our findings. At a fine scale, sturgeon chub have an affinity for gravel substrates (Davis & Miller, 1967; Reigh & Elsen, 1979; Welker & Scarnecchia, 2004; Werdon, 1992), and our study determined that the probability of their presence increased as the percentage of gravel increased in the White and Little White rivers. Bottom velocity (Everett et al., 2004; Welker & Scarnecchia, 2004) and depth (Everett et al., 2004) have also been determined to be important predictors of sturgeon chub presence. Our results suggest sturgeon chub use shallower depths and slower bottom velocities than what has been reported from mainstem studies. For example, in two different segments of the Missouri River, sturgeon chub were reported at mean depths of 4.8 and 8.7 m as well as bottom velocities of 0.7 and 1.5 m/s (Everett et al., 2004). Comparatively, we documented sturgeon chub at mean depths ranging from 0.25 to 0.49 m and bottom velocities ranging from 0.32 to 0.34 m/s. Understandably, this is likely due to differences in habitat availability influenced by variation in geomorphic and hydrological processes between mainstem and tributary rivers. Further, the influence of depth and velocity as environmental predictor variables may vary depending on other factors, such as turbidity. For example, Gould (1994) found that sturgeon chub used shallow areas when waters were highly turbid and deeper areas in less‐turbid waters in Montana. This is consistent with our results and may explain why a strong relationship with depth was found in our regression model for the Cheyenne River, but not the much more turbid White River. Our findings highlight the need to better understand the habitat requirements at different spatial scales of sturgeon chub in tributary rivers to ensure appropriate conservation measures for the species.

Impoundments are known to impact fish assemblage structure by reducing native species diversity and abundance and introducing nonnative species (Berry & Young, 2001; Galat et al., 2005; Hoagstrom et al., 2007; Pegg et al., 2003; Quist et al., 2005). Fragmentation and turbidity reductions combined with the introduction of nonnative sight‐feeding predators are considered detrimental to native chubs in altered rivers (Rahel & Thel, 2004; Werdon, 1992), which could be another factor limiting the upstream extent of sturgeon chub and other native species in the Cheyenne River. Hoagstrom et al. (2007) suggested that predation by smallmouth bass could have contributed to the absence of other native broadcast spawning minnows, including flathead chub and *Hybognathus* spp., in the Cheyenne River within the 70 river kilometres below Angostura Dam. The influences of major tributaries that join the middle Cheyenne River further downstream from Angostura Dam mitigate the abiotic and biotic influences of the impoundment (Hoagstrom et al., 2007), thus creating conditions that are more suitable for sturgeon chub and other native, large‐river species.

We found that reaches with sturgeon chub presence had similar fish communities. Previous studies have found sturgeon chub to be associated with flathead chub (Gould, 1994; Grisak, 1996; Quist et al., 2004; Stewart, 1981; Welker & Scarnecchia, 2004; Werdon, 1992; Wildhaber et al., 2012) and *Hybognathus* spp. (Quist et al., 2004; Stewart, 1981; Welker & Scarnecchia, 2004), which aligns with our results. Flathead chub possess similar life‐history strategies to sturgeon chub and are also largely absent from the highly altered mainstem Missouri River in South Dakota (Hesse et al., 1993; Quist et al., 2004; Welker & Scarnecchia, 2004). The association that we found between sturgeon chub and sauger could be tied to predator–prey interactions in these rivers, as sturgeon chub were identified as an important prey source for sauger in the Missouri River (Gardner & Berg, 1982; Rahel & Thel, 2004; Weitzel, 2002). Particularly, on a temporal scale, shifts in overall fish community assemblages may also contribute to the differences in sturgeon chub composition between our study and historical surveys (Fryda, 2001; Jones, 2018). For example, if historically there were more sauger, then sturgeon chub composition would possibly be lower. Interestingly, Galat et al. (2005) reported sauger declining in recent years in the Missouri River mainstem. Nonetheless, species associations may serve as useful indicators of suitable conditions for the persistence of sturgeon chub and other minnows with similar life‐history strategies and offer a broader ecological context for understanding their distribution in tributary systems.

## 5 CONCLUSION

Sturgeon chub populations are currently persisting in Missouri River tributaries of South Dakota. The longer, unfragmented river lengths with relatively natural hydrological regimes and high habitat complexity that characterize these rivers appear to provide more suitable conditions for the persistence of sturgeon chub. However, sturgeon chub may be limited longitudinally in these tributaries during times of drought, creating fragmented habitats and loss of river connectivity that could affect spawning and recruitment. Although droughts may have occurred historically, climate change may lead to more severe drought conditions in western South Dakota by 2050 (Milly et al., 2005; Perkin et al., 2015). Sturgeon chub are a short‐lived species, making them susceptible to significant population changes over short periods (Albers, 2014a; Albers, 2014b; NGPC, 2010; Pflieger, 1997; SDGFP, 2006; Wilde & Durham, 2008). Therefore, more frequent monitoring efforts for these species are needed to assess temporal changes in their population levels. These tributaries also provide opportunities for future research to fill in the knowledge gaps regarding many unknown aspects of sturgeon chub, including but not limited to completing their life‐history cycle via reproduction, feeding habits and physiology. In particular, our observations of larger, spawning adults upstream and young‐of‐the‐year individuals downstream suggest that reproduction is occurring within these systems and may involve seasonal movements. The presence of age 0+ individuals in the lower reaches further implies a protracted spawning period, potentially involving multiple spawning bouts within a single season. Targeted studies on spawning timing, habitat use and juvenile dispersal would provide critical insights into the life history of sturgeon chub and inform conservation planning. Additionally, future studies could examine sturgeon chub habitat selectivity and explore space use in the context of utilization distribution to gain better insights into where to prioritize habitat conservation efforts. Based on our results, conservation efforts should be placed on these tributaries and other large, relatively unaltered rivers that harbour imperilled, large‐river species that have been displaced by mainstem alterations.

## AUTHOR CONTRIBUTIONS

All authors participated in visualization, validation and writing – review and editing. Conceptualization: Mitchell R. Magruder and Mark A. Pegg; data curation: Mitchell R. Magruder; investigation: Mitchell R. Magruder and Mark A. Pegg; methodology: Mitchell R. Magruder and Mark A. Pegg; resources: Mitchell R. Magruder and Mark A. Pegg; project administration: Mark A. Pegg; funding acquisition: Mark A. Pegg; writing – original draft preparation: Mitchell R. Magruder, Jenna P. Ruoss and Mark A. Pegg.

## FUNDING INFORMATION

Funding for this project was provided through a State Wildlife Grant with South Dakota Game, Fish and Parks (Project T‐89‐R1) and the Nebraska Agricultural Experiment Station with funding from the Hatch Act (Project NC 1189) through the United States Department of Agriculture National Institute of Food and Agriculture.

## Supporting information


**Table S1.** Species codes and common names for nonmetric multidimensional scaling (NMDS) species ordination.
