# Supplementary material for: Distributional patterns and habitat associations of sturgeon chub in western Missouri River tributaries of South Dakota
Source: J Fish Biol. 2026 Mar 29;108(5):1565–81. doi: 10.1111/jfb.70414 (PMC13273089; doi:10.1111/jfb.70414)
Supplement: Supplementary file 1 — Table S1. Species codes and common names for nonmetric multidimensional scaling (NMDS) species ordination. [file JFB-108-1565-s001.pdf]

SUPPORTING INFORMATION

Table 1.

Species Code	Common Name
BMBF	Bigmouth Buffalo
BKBH	Black Bullhead
BKCP	Black Crappie
BLGL	Bluegill
CNCF	Channel Catfish
CARP	Common Carp
CKCB	Creek Chub
ERSN	Emerald Shiner
FHMW	Fathead Minnow
FHCB	Flathead Chub
FWDM	Freshwater Drum
GDEY	Goldeye
GNSF	Green Sunfish
HBNS	*Hybognathus* spp.
LMBS	Largemouth Bass
LNDC	Longnose Dace
NTPK	Northern Pike
PKLF	Plains Killifish
PTMW	Plains Topminnow
RDSN	Red Shiner
RVCS	River Carpsucker
RKBS	Rock Bass
SNSN	Sand Shiner
SGER	Sauger
SHRH	Shorthead Redhorse
SNGR	Shortnose Gar
SMBS	Smallmouth Bass
SMBF	Smallmouth Buffalo
STCT	Stonecat
WLYE	Walleye
WTBS	White Bass
WTSK	White Sucker
YLBH	Yellow Bullhead
YWPH	Yellow Perch
